# Gaining Insight into Teenagers’ Experiences of Pain after Laparoscopic Surgeries: A Prospective Study

**DOI:** 10.3390/children11040493

**Published:** 2024-04-20

**Authors:** Mihaela Visoiu, Jacques Chelly, Senthilkumar Sadhasivam

**Affiliations:** 1 UPMC Children’s Hospital of Pittsburgh, Department of Anesthesiology and Perioperative Medicine, University of Pittsburgh Medical Center, 4401 Penn Avenue, Pittsburgh, PA 15224, USA; sadhasivams@upmc.edu; 2 UPMC Shadyside Hospital, Department of Anesthesiology and Perioperative Medicine, University of Pittsburgh Medical Center 5230 Center Ave, Pittsburgh, PA 15232, USA; chelje@anes.upmc.edu

**Keywords:** teenager postoperative pain, visual analog pain scale, pain behavior, pain catastrophizing thoughts, anxiety, mood, laparoscopic surgeries

## Abstract

There is an anecdotal impression that teenage patients report exaggerated postoperative pain scores that do not correlate with their actual level of pain. Nurse and parental perception of teenagers’ pain can be complemented by knowledge of patient pain behavior, catastrophizing thoughts about pain, anxiety, and mood level. Two hundred and two patients completed the study—56.4% were female, 89.6% White, 5.4% Black, and 5% were of other races. Patient ages ranged from 11 to 17 years (mean = 13.8; SD = 1.9). The patient, the parent, and the nurse completed multiple questionnaires on day one after laparoscopic surgery to assess patient pain. Teenagers and parents (r = 0.56) have a high level of agreement, and teenagers and nurses (r = 0.47) have a moderate level of agreement on pain scores (*p* < 0.05). The correlation between patient APBQ (adolescent pain behavior questionnaire) and teenager VAS (visual analog scale) and between nurse APBQ and teenager VAS, while statistically significant (*p* < 0.05), is weaker (r range = 0.14–0.17). There is a moderate correlation between teenagers’ pain scores and their psychological assessments of anxiety, catastrophic thoughts, and mood (r range = 0.26–0.39; *p* < 0.05). A multi-modal evaluation of postoperative pain can be more informative than only assessing self-reported pain scores.

## 1. Introduction

Laparoscopic surgeries are increasingly prevalent for various medical conditions among children. Despite the numerous benefits of these minimally invasive procedures, assessing and managing postoperative pain remains a significant concern [1,2,3]. Adolescence signifies physical transformation, ongoing cognitive and psychological development, and the transition from childhood to adulthood. Assessing postoperative pain in teenagers poses a challenge in pediatric care [4]. Several factors, such as individual pain thresholds, prior pain experiences, anxiety levels, and support systems, can contribute to teenagers’ pain perception [5]. There is a common observation, both anecdotally and clinically, that teenage patients sometimes report exaggerated levels of postoperative pain that may not align with the actual severity [6]. Pain, as experienced by teenagers, is subjective and valid whenever they express it. Huguet et al. suggested that self-reported pain should be complemented by observation and knowledge of the context [7]. Despite potential disparities in our subjective interpretations of reported pain and a lack of precise explanations for perceived exaggerated pain scores, our paramount goal is to alleviate our patients’ suffering. The repercussions of untreated pain can be severe, leading to behavioral issues and laying the groundwork for future pain experiences [5].

On the other hand, managing postoperative pain can inadvertently contribute to prolonged opioid use and misuse [8]. Pediatric nurses administer more pain medications to the children who vocalize their pain [7,9]. Clinical interpretation of self-reported pain scores in teenagers can be difficult. Understanding the reasons behind our suspicion that teenage patients may not consistently report pain accurately is intricate and likely extends beyond mere perception [6].

It is essential to delve into the pain perception of caregivers (both parents and nurses), as well as the pain behavior and psychological factors of teenagers, which can impact their self-reported pain. In clinical practice, patient self-reporting of pain is standard [10]. The visual analog scale (VAS) is a widely recognized instrument for evaluating children’s postoperative pain and is highly endorsed for self-reported acute pain assessments [11]. While this scale may occasionally underestimate or overestimate actual pain levels, establishing a framework to correlate patient–parent and patient–nurse pain scores, pain scores with pain behavioral observations, and physiological parameters can enhance our comprehension of teenage self-reported pain.

The dynamics between patients, parents, and nurses hold considerable importance during the postoperative phase. The bond between parents and children is deeply intertwined [12,13]. The child’s display of pain can evoke distress in parents, who often act as secondary reporters of their children’s pain, potentially leading to unintended outcomes. While healthcare professionals sometimes underestimate patients’ pain [14], the nurse’s perspective can significantly influence pain management, particularly in complex scenarios. Nevertheless, the reliability of these relationships (teenager–parent, teenager–nurse) in evaluating postoperative pain within the teenage demographic remains to be definitively established.

Communication about health issues often presents challenges during adolescence. Pain expressions serve as valuable indicators of teenagers’ reactions to pain, and both parents and nurses can rely on the patient’s pain behavior to evaluate the adolescent’s pain experience [15]. Regrettably, there remains to be more consensus regarding the alignment between observed pain behavior and self-reported pain intensity. Nevertheless, integrating this measure into the assessment process can aid in navigating the intricate task of evaluating postoperative pain in teenagers.

In addition to self-reported pain and pain behavior, a comprehensive assessment should encompass considerations of anxiety levels, catastrophic thinking about pain, and mood. Teenage patients’ anxiety levels are known to correlate with the severity of pain [16,17,18]. However, studies investigating the relationship between stress and acute postoperative pain in teen patients are scarce, highlighting the importance of evaluating trait anxiety as part of a comprehensive assessment battery. Catastrophic thinking regarding one’s pain is associated with heightened attention to pain [19,20,21,22,23]. Teenagers naturally experience a range of moods and mood swings during adolescence. In the postoperative period, an adolescent patient may transition rapidly from feelings of happiness and excitement to those of distress and fear. Yet, the relationship between a teenager’s mood and postoperative pain has not been thoroughly explored.

We suspected that teenage patients may sometimes report pain scores that appear exaggerated and do not align with anticipated postoperative pain levels. To investigate this theory, we conducted the following steps: (1) gathered self-reported pain scores from teenage patients on the first day after surgery, when expected pain levels are typically mild to moderate; (2) collected feedback (VAS pain scores) from the patient’s parents and nurses regarding the reported pain scores; (3) obtained information from parents and nurses about the patient’s pain-related behavior to assess the agreement between subjective pain scores and objective pain behavior; and (4) gathered psychological factors that might be linked to pain perception and potentially amplify the sensation of pain.

The primary objectives of this investigation were to (1) to evaluate the concordance between the pain assessments provided by parents and nurses and the pain scores reported by teenage patients, anticipating a small-to-moderate correlation; (2) to determine correlations between reported pain scores and evaluations of teenage pain behavior given by both parents and nurses, with an expected small-to-moderate correlation; and (3) to identify psychosocial factors such as anxiety, catastrophic thoughts, and mood that could potentially influence teenage pain perception, with an expected moderate-to-high correlation. In essence, this study aimed to answer the following overarching question: When a teenage patient’s self-reported pain appears higher than expected, can the combined assessments from nurses and parents regarding the patient’s postoperative pain scores, along with observations of the patient’s pain behavior, catastrophic thoughts, anxiety, and mood, help healthcare providers ascertain whether the self-reported pain score accurately represents the patient’s pain experience?

## 2. Materials and Methods

### 2.1. Study Design

This research was carried out at UPMC Children’s Hospital of Pittsburgh (CHP) from December 2012 to August 2014 and received approval from The University of Pittsburgh Institutional Review Board. It was registered on www.clinicaltrials.gov, accessed on 20 May 2023, (NCT 017555065) in December 2012. Participants were recruited from CHP based on a review of medical records and a medical interview with the patient and parent on the surgery day. Among the 2241 patients who underwent laparoscopic procedures, including robotic cholecystectomies, at CHP during the study period, 959 were excluded due to their procedure type, 623 were outside the target age range, 78 were excluded due to unavailability of the study team, and 351 did not meet the inclusion/exclusion criteria upon medical record review. Out of the 230 patients approached for the study, 6 were ineligible, 3 refused to participate, two did not complete consent, 6 were discharged before completing questionnaires, 3 parents were unavailable on postoperative day (POD) 1, 3 withdrew consent on POD 1, and 3 procedures were converted to open surgeries. Three patient/parent/nurse units did not complete all study assessments. Ultimately, 202 patients (along with their parents and nurses) completed all study procedures (Figure S1).

Patients included in the study were aged between 11 and 17 years old, scheduled for elective or emergent laparoscopic surgeries, and required overnight admission. Exclusion criteria encompassed chronic pain conditions, non-English-speaking families, cognitive impairment history, developmental delay, psychiatric medical history (except for ADD and ADHD), positive pregnancy tests, drug use (including marijuana), medication with certain opioids or psychiatric drugs, surgical complications, discharge on the day of surgery, conversion of laparoscopic surgeries to open procedures, and lack of parental availability for questionnaire completion.

The surgical procedures conducted included various laparoscopies, such as laparoscopic appendectomy (132 cases), cholecystectomy (49 cases, including robotic cholecystectomies in 5 cases), diagnostic laparoscopy (11 cases), nephrectomy (1 case), oophorectomy (3 cases), cystectomy and cyst drainage (3 cases), Heller myotomy (1 case), and laparoscopic Meckel resection (2 cases). The administration of anesthesia was not standardized by the study protocol, affording discretion to the anesthesiologist and acute pain service regarding techniques and medication choices. Anesthesia was predominantly general (97 cases), either solely or in combination with regional anesthesia techniques (105 patients), such as rectus sheath blocks (101 patients), transversus abdominis plane blocks (47 patients), paravertebral nerve blocks (2 patients), or a combination of rectus and transversus abdominis blocks (45 patients). Medications for general anesthesia included propofol, midazolam, fentanyl, morphine, hydromorphone, ketamine, and rocuronium, while regional anesthesia involved the use of ropivacaine. Additionally, the same local anesthetic was administered in the surgical wound if a block was not performed. Postoperative pain management comprised morphine, hydromorphone, oxycodone, acetaminophen, and ibuproferen. After surgery, opioids were administered for 169 patients (83.7%).

### 2.2. Study Protocol

Informed consent was obtained from patients or their guardians on the day of surgery before the procedure. Patient demographic data were collected from medical records, and pain scores, pain behavior, anxiety levels, catastrophic thoughts, and mood levels were assessed using various questionnaires. For data collection, research assistants visited patients, parents, and nurses on the first-day after surgery, ensuring the blinding of participants from each other’s scores. Patients completed questionnaires, including VAS and psychological assessments, while a family member (preferably the mother) completed related questionnaires (pain scores and behavior), and nurses completed the same assessments independently in the first 24 h after the surgeries were completed. All assessments were conducted simultaneously.

### 2.3. Questionnaires

#### 2.3.1. Visual Analog Scale (VAS)

The visual analog scale (VAS) consists of a horizontal line measuring 100 mm. Descriptors are positioned at each end of the line: “no pain” at one extreme and “the worst pain imaginable” at the other (100 mm mark). Patients indicate their pain level by marking on the line. Additionally, the nurse and the parent utilize a VAS, each indicating their perception of the teenager’s pain intensity.

#### 2.3.2. Adolescent Pain Behavior Questionnaire (APBQ) [15]

This instrument is a parent-reported assessment of pain expressions in adolescents aged 11 to 19 [15]. It comprises 23 items and demonstrates strong internal consistency (alpha = 0.93). Parents evaluate each pain behavior on a scale ranging from 0 (never) to 5 (almost always), resulting in a total score ranging from 0 to 115 [15]. Lynch-Jordan et al. conducted initial reliability and validity assessments involving 138 parent–adolescent pairs to establish their psychometric properties [15].

#### 2.3.3. Pain Catastrophizing Scale for Children (PCS-C) [24]

The pain catastrophizing scale for children (PCS-C) is a 13-item questionnaire adapted from the adult version. This adaptation involved rephrasing one item, simplifying the rating scale, and prefacing each item with the stem “When I am in pain…” [24]. Children use a five-point scale (0 = “not at all”; 4 = “extremely”) to rate the frequency of experiencing various thoughts and feelings during pain episodes. The PCS-C comprises three subscales: (1) rumination (e.g., “… I keep thinking about how much it hurts”.); (2) magnification (e.g., “… I wonder whether something serious might happen”.); and (3) helplessness (e.g., “… there is nothing I can do to reduce the pain”.). Construct and predictive validity have been demonstrated [24]. The PCS-C provides a total score ranging from 0 to 52 and subscale scores for rumination, magnification, and helplessness. The scale exhibits excellent reliability, with a Cronbach’s alpha of 0.92; subscale alphas in the current sample ranged from 0.68 to 0.88.

#### 2.3.4. State–Trait Anxiety Inventory for Children (STAIC S—Anxiety) [25]

On postoperative day 1 (POD 1), participants completed the state version of the state–trait anxiety inventory for children to assess teenage anxiety levels. The STAIC S—Anxiety scale comprises 20 statements prompting teenagers to indicate their current feelings (e.g., “I feel…”) by selecting one of three options that best describes their state (e.g., “very calm”, “calm”, or “not calm”). Scores on this scale range from 20 to 60. In the current study, the alpha reliability of the STAIC S—Anxiety scale was determined to be 0.90.

#### 2.3.5. Brief Mood Introspection Scale (BMIS) [26]

The brief mood introspection scale (BMIS) comprises 16 mood adjectives, with two selected from each of the eight mood states (happy, loving, calm, energetic, anxious, angry, tired, and sad) [26]. Participants were instructed to rate how well each adjective described their current mood using a 4-point scale ranging from 1 (“definitely do not feel”) to 4 (“definitely feel”). Positive adjectives were assigned positive values, while negative adjectives were assigned negative values, resulting in a total score ranging from −24 to +24. In the current study, the scale demonstrated moderate reliability, with a Cronbach’s alpha of 0.8.

### 2.4. Statistical Analyses

The basic descriptive statistics (including means, medians, and standard deviations) were used to describe the measurements. Boxplots, scatter plots, and q–q plots examined the distributional assumptions for all variables of interest. The proposed analysis involved the computation of Pearson’s and Spearman’s correlation coefficient between the VAS pain scores for teenagers and each of the VAS pain scores for the parent and nurse, between the VAS pain scores for teenagers and the pain behavior scores reported by the nurse and family, and between the VAS pain scores and the psychosocial factors. The correlations were considered weak if values were 0.23 to <0.30, moderate if 0.30 to <0.50, and high if ≥0.50. 

#### Sample Size and Power

For a small correlation of 0.23, a sample size 206 was required to achieve 80% power using a two-sided hypothesis test with a significance level of 0.0125 (Bonferroni correction for all four comparisons, 0.05/4 = 0.0125). Anticipating that the sample size is 206 with three observations (i.e., teenager, parent, and nurse), the study was powered (80%) to detect an intraclass correlation coefficient of 0.10 using an F-test with a significance level of 0.05. To determine inter-rater reliability between two observations (e.g., teenager versus parent), the study achieves 80% power to detect an intraclass correlation of 0.19 using an F-test with a significance level of 0.025 to account for multiple comparisons. Outcome measure scores and Pearson and Spearman correlation coefficients were calculated using SAS software Version 9.3 of the SAS System for Windows, Copyright ©2002–2010 SAS Institute Inc., Cary, NC, USA.

## 3. Results

**Figure 1:**
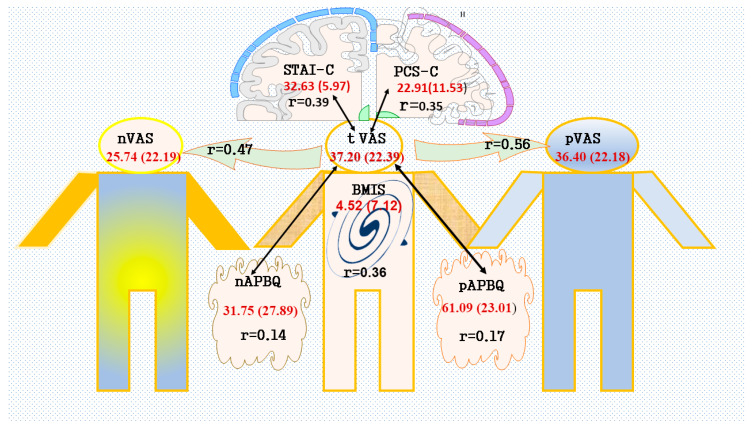
Perioperative variables parents and nurses report influencing teenagers’ postoperative pain perception, mood, and catastrophic thoughts. Continuous measures are presented as mean ± standard deviation. r is the correlation coefficient; VAS is the visual analog scale; BMIS is the brief mood introspection scale; STAI-C is the state–trait anxiety inventory for children; PCS-C is the pain catastrophizing scale for children; APBQ is the adolescent pain behavior questionnaire; t is teenager; n is nurse; p is parent. The correlations were considered weak if values were 0.23 to <0.30, moderate if 0.30 to <0.50, and high if ≥0.50. Figure composed using Motifolio, Inc. diagrams.

Two hundred and two (202) patients (mean age = 13.8 years; SD 1.9) were included in the final analysis. Patient characteristics are presented in Table 1. Descriptive statistics of the outcome measures are reported in Table 2, and the results are summarized in Figure 1.

Teenagers and parents (r = 0.56) have a high level of agreement on pain scores (Table 3), and teenagers and nurses (r = 0.47) have a moderate level of agreement on pain scores (*p* < 0.05) (Table 4). The correlation between APBQ and VAS and between APBQ and VAS, while statistically significant (*p* < 0.05), is very weak (r range = 0.14–0.17). Parent perception of child pain was influenced only by facial (r = 0.172; *p* < 0.05) and verbal expression (r =0.164; *p* < 0.05). Nurse perception of patient pain was influenced by facial expression (ρ = 0.172; *p* < 0.05), verbal expression (ρ = 0.143; *p* < 0.05), and child behavior (ρ = 0.153; *p* < 0.05) (Table 3 and Table 4).

There is a moderate correlation between teenagers’ pain scores and their psychological assessments of anxiety, catastrophic thoughts, and mood (r range = 0.26–0.39; *p* < 0.05) (Table 5).

Given developmental maturation in teenagers, we further evaluated age and sex subgroups separately. Still, we found the same pattern of correlation coefficients among the 11–13-year-old subgroup (N = 88) (Supplementary Tables S1–S3), the 14–17-year-old subgroup (N = 114) (Supplementary Tables S4–S6), and the female (N = 114) (Supplementary Tables S7–S9) and the male (N = 88) (Supplement Tables S10–S12) subgroups as we found in the study population as a whole.

## 4. Discussion

Consistent with prior studies, we have observed that assessing and managing pain in teenage patients is intricate and presents considerable hurdles for healthcare professionals [3]. There is a heightened emphasis on achieving optimal postoperative pain relief, leading medical providers to often administer opioid medication to teenagers reporting a numeric rating scale (NRS) pain score of 4 or higher. However, it is not uncommon for us to perceive that teens may sometimes exaggerate their pain scores. Therefore, there is a pressing need to gain a deeper understanding of self-reported pain scores and verify their credibility.

Various self-reported pain scales are utilized to assess postoperative pain, including the numeric rating scale (NRS), visual analog scale (VAS), and verbal rating scale (VRS). The VAS, in particular, is commonly employed due to its strong reliability, validity, responsiveness, acceptability, cost-effectiveness, and metric properties [27,28,29,30,31]. It is considered suitable for children aged eight and above [32]. From a statistical perspective, the VAS tends to be more robust and sensitive to changes than other self-reported scales [27,28,29,30]. However, some individuals may find it challenging to translate their subjective perception of pain intensity into a mark on a straight line. This can lead to exaggeration, minimization, or unrealistic representations of pain [1,33]. Moreover, VAS pain scores offer an incomplete depiction of the pain experience, prompting the need to explore other factors to comprehend the multidimensional nature of postoperative pain. Huguet et al. proposed that self-reported pain assessments should be supplemented with observation and contextual knowledge [7]. Such an approach can prove invaluable, particularly when assessing the credibility of self-reported pain.

Children and adolescents rely on their parents for care, and parents bear the responsibility for their postoperative well-being, leading to a potential interconnectedness between parental involvement and pain scores [12,13,34]. Parents, being familiar with their child’s typical behavior, are well positioned to recognize pain-related behaviors, while nurses’ knowledge can significantly influence pain management for the child. Research by van Dijk et al. highlighted that pediatric patients’ visual analog scale (VAS) pain scores exhibit varying correlations (ranging from 0.23 to 0.85) with scores reported by different caregivers, including parents, nurses, researchers, and physicians [35]. Additionally, van Dijk and Khin Hla et al. discovered that children aged 3 to 11 and their parents moderately agree on pain scores (r = 0.113) [29,30]. However, it is important to note that these findings may not directly apply to teenage patients. Nonetheless, our study revealed substantial agreement (r = 0.56) between teenagers and their parents regarding pain scores.

Disputes regarding the alignment between patients’ and healthcare providers’ pain assessments persist. Seers et al. went as far as to suggest discontinuing studies comparing such scores due to the tendency of professionals to underestimate patients’ pain [14]. Both Seers et al. and Khin Hla et al. observed a pattern where nurses tended to report lower scores (0 [0–2]) compared to parents (2 [1–4]) and pediatric patients (2 [0–4]) [14,36]. Despite these observations, pediatric studies have yet to conclusively establish healthcare providers’ ability to gauge postoperative pain accurately. The extent of underestimation likely hinges on the severity of the patient’s pain and warrants further exploration. Healthcare providers’ perspectives play a pivotal role in ensuring appropriate pain management. Nurses may hold perspectives that differ significantly from those of patients, with their pain scores influenced by factors such as their medical knowledge regarding surgical tissue damage, vital signs, relationships with the child and family, time spent with the child, training [14], experience, and patient rapport [37]. While our study corroborated that nurses tended to report lower scores for mild pain than patients, it also demonstrated alignment between nurses, parents, and teenagers. Furthermore, the degree of underestimation was minimal (25.5 ± 22.1 vs. 37.0 ± 22.3) and is unlikely to significantly contribute to the undertreatment of pain.

The most appropriate type of pain behavior for evaluating teenagers’ postoperative pain management remains uncertain. Pain behavior encompasses verbal and non-verbal expressions, including the subjective interpretation of facial expressions, body posture, gestures, level of activity, and breathing patterns [2,38]. It can be under either conscious or unconscious control [39]. Anecdotal observations, as well as findings from other researchers, suggest that many teenage patients with postoperative pain may report high pain scores while appearing relaxed and engaging in activities such as playing games or texting friends, which may indicate little to no pain [2]. However, our literature review uncovered only a limited number of small-scale pediatric studies comparing self-reported visual analog scale (VAS) pain scores with observed pain behavior, with variations in pain behavior investigated across studies. In a meta-analysis encompassing 29 studies (with adult studies contributing to 82% of the data), considerable variability was noted among the studies, albeit with a moderately positive association overall (z = 0.26) [40]. This association is more likely to be significant when the individual under study experiences acute pain (z = 0.35) and when self-reported pain intensity data are collected promptly after observing pain behavior (z = 0.40) [40].

A 23-item pain behavior questionnaire, known as the APBQ, was employed to evaluate chronic pain in teenage patients [15]. In a study involving 138 parent–adolescent dyads (aged 11–19 years), no significant relationship was identified between parent-reported pain behaviors and adolescent-reported chronic pain intensity [15]. However, a slight yet notable correlation emerged between parents’ estimations of their adolescent’s pain intensity and their reports of observed pain behaviors (r = 0.25). While the APBQ was not specifically validated for use with teenagers experiencing acute postoperative pain, we deemed this questionnaire more suitable for our patient population compared to other pain behavior scales available. It is worth noting that, in contrast to Lynch-Jordan’s study [15], we identified a very weak but statistically significant correlation between reported teenage pain scores and parent-reported pain behaviors (r = 0.16). These findings offer some insight into our observations that teenagers’ pain behaviors may not always align closely with the reported pain intensity.

It is important to note that the association between nurse pain scores and teenage pain behavior was found to be minimal in our study. Additionally, the “behavior” subscale of the APBQ did not emerge as a significant predictor of teenage pain scores when parents completed the questionnaire. Understanding why the correlation between nurse/parent pain scores and teenage pain behavior is so weak presents a challenge. Throughout our study, pain scales were completed independently, with both the nurse and parent being aware of the child’s pain behavior but unaware of the child’s self-reported pain rating. However, given the frequent pain assessments conducted by both the nurse and parent during the hospital stay, it is plausible that they struggled to separate their perception of the child’s pain behavior from what the child was verbally communicating (pain scores reported before questionnaire completion).

It is worth noting that the association between nurse-reported pain scores and teenage pain behavior was minimal in our study. Additionally, when parents completed the APBQ, the “behavior” subscale did not emerge as a significant predictor of teenage pain scores; understanding why the correlation between nurse/parent pain scores and teenage pain behavior is weak presents a challenge. Throughout our study, pain scales were completed independently, with both the nurse and parent being aware of the child’s pain behavior but unaware of the child’s self-reported pain rating. However, given the frequent pain assessments conducted by both the nurse and parent during the hospital stay, it is conceivable that they found it difficult to distinguish between the child’s pain behavior and what the child verbally communicated (pain scores reported before questionnaire completion).

Adolescent patients undergoing surgery may exhibit varying perceptions of their subjective postoperative pain, often accompanied by poor psychological adjustment to acute postoperative pain, characterized by heightened levels of anxiety and increased attention to pain-related catastrophizing thoughts. Furthermore, fluctuations in mood can influence reported pain scores. This study, while essentially reaffirming expected associations between pain scores and psychological factors, including psychological interventions, holds promise for effectively managing and alleviating postoperative pain [41]. Notably, teenagers experiencing chronic pain have been found to exhibit higher levels of depressive symptoms and more pronounced pain-catastrophizing thoughts, which significantly correlate with parent-reported pain behavior [15]. Similar findings were observed for teenagers experiencing acute postoperative pain and engaging in catastrophizing behaviors. This study represents the first attempt to explore the relationship between mood and postoperative pain. During the postoperative period, teenage patients may experience rapid mood swings, transitioning from feelings of happiness and excitement to feelings of distress and apprehension. Interestingly, our findings revealed a moderate negative correlation between mood and pain levels.

There are some limitations to this study.

Initially, it is important to note that our study’s reported mean pain level was mild (37.0 ± 22.3), and our investigation did not specifically target teenagers experiencing severe or “unreal” (exaggerated, and not align with the actual severity pain). It is crucial to acknowledge that teenagers’ psychological flexibility may influence psychological assessments conducted the day after surgery. Unfortunately, our study lacked baseline measurements of psychosocial factors. Consequently, whether including baseline psychological measurements would alter our findings remains to be seen. Furthermore, we must emphasize that while we provide correlational values between various parameters, we are unable to provide recommendations on how to utilize our findings to guide opioid dosing in the postoperative period. Additionally, we are cautious about relying solely on pain behavior as a measure of postoperative pain. In challenging situations, the absence of observable signs of pain behavior does not necessarily indicate the absence of pain in the patient.

Additionally, it is important to note that we specifically requested mothers to complete the questionnaires, given their frequent role as primary caregivers. However, we acknowledge that this approach may introduce bias, as mothers’ responses to child pain may differ from those of fathers [42,43,44]. Nonetheless, previous research has indicated that mothers and fathers do not significantly differ in their levels of catastrophizing and trait anxiety [44,45]. It is worth highlighting that in our study, mothers were predominantly available for participation, rather than fathers.

Finally, it is crucial to recognize that our findings pertain specifically to teenagers experiencing mild-to-moderate postsurgical pain and may not be suitable for generalization to individuals undergoing more painful surgical procedures. Conducting a study exclusively focusing on such patients would be impractical and ethically challenging. Nevertheless, despite this limitation, our study sheds light on several modalities for assessing pain in ambiguous and complex situations.

Need to answer questions and future research. 

Adolescents often experience feelings of hopelessness, and it is essential not to disregard their reported pain scores. In challenging circumstances, it becomes crucial to identify potential pain sources, including surgical and non-pain-related distress, and respond accordingly. While our study confirms expected levels of agreement between patients and multiple providers and between pain scores and psychosocial factors, it lays a vital foundation for future research endeavors. Developing a risk stratification algorithm that could pinpoint strategies to mitigate pain severity and reduce the necessity for postoperative opioid administration marks just the beginning. Several key observations should be considered when assessing a patient experiencing severe pain that healthcare providers perceive as exaggerated or unreal: (1) parent-proxy pain scores may underestimate teen pain scores, indicating a discrepancy between the severity perceived by the patient and the parent (i.e., patient-severe but parent-moderate); (2) nurse proxy pain scores may similarly underestimate teen pain scores (i.e., patient-severe but nurse-mild); (3) evaluating pain behavior, such as a relaxed body, ease of movement, absence of verbal complaints, stable vitals, and engagement in enjoyable activities like video games, may suggest lower pain severity; (4) assessing the teen’s anxiety level, with signs of calmness, pleasantness, cheerfulness, relaxation, happiness, and satisfaction suggesting a lower anxiety level; (5) considering the teen’s catastrophizing thoughts, with the absence of worrying or fear of pain indicating lower levels of catastrophizing thoughts or feelings; (6) evaluating the patient’s mood, with a lively, happy, caring, calm, content, loving, and active demeanor suggesting a positive mood. Additionally, employing appropriate questionnaires to identify psychological distress can offer an objective means of assessing pain and psychological factors in such scenarios. However, further studies are warranted to investigate the practicality and effectiveness of implementing these strategies postoperatively and their impact on satisfaction with pain control.

## 5. Conclusions and Clinical Implications

To summarize, our study indicates that a multi-modal approach to assessing postoperative pain, incorporating pain scores from teenagers, parents, and nurses, along with evaluations of pain behavior, pain catastrophizing thoughts, anxiety, and mood, can provide richer insights compared to relying solely on self-reported pain scores. Given the growing concerns regarding opioid addiction and associated risks, it is imperative to reduce opioid use in the postoperative period. Integrating parent and nurse perspectives into pain assessment and considering psychological factors are crucial aspects of practice. Alongside analgesics, psychological interventions like preparation, education, distraction, imagery, and video games, as well as managing expectations, should be contemplated. Truncal blocks may enhance pain control following laparoscopic surgeries and warrant consideration. However, further research is necessary to comprehend teenagers’ postoperative experiences and explore potential interventions fully.

## Figures and Tables

**Table 1:** Patient characteristics.

	N = 202
Sex	Male 88 (43.6%) Female 114 (56.4%)
Race	White 181 (89.6%) Black 11 (5.4%) Other 10 (5.0%)
Age (years)	13.8 ± 1.9 (11.0–17.0)
Weight (kg)	59.9 ± 17.2 (20.1–125.5)
Height (cm)	161.7 ± 10.3 (131.0–192.0)
BMI	22.9 ± 5.4 (12.2–43.4)
Diagnosis of ADHD	18 (8.9%)
Use of ADHD medication	7 (3.5%)

Note: Continuous measures are presented as mean ± standard deviation and range of values. N is the number of patients. kg are kilograms; cm are centimeters; BMI is body mass index; ADHD is attention deficit hyperactive disorder.

**Table 2:** Outcome measures.

	N = 202
**Teenage patient responses**	
VAS	37.0 ± 22.3 (0.0–91.0)
BMIS	4.6 ± 7.1 (−18.0–21.0)
STAI-C	32.5 ± 5.9 (21.0–51.0)
PCS-C	22.9 ± 11.6 (1.0–50.0)
Rumination	10.0 ± 4.2 (0.0–16.0)
Magnification	3.8 ± 2.8 (0.0–12.0)
Helplessness	9.1 ± 5.9 (0.0–23.0)
**Parent responses**	
VAS	36.3 ± 22.2 (0.0–91.0)
APBQ	61.0 ± 23.1 (7.0–113.0)
Facial	16.2 ± 7.2 (0.0–34.0)
Verbal	22.5 ± 9.9 (0.0–46.0)
Behavioral	22.3 ± 8.7 (2.0–45.0)
**Nurse responses**	
VAS	25.5 ± 22.1 (0.0–83.0)
APBQ	31.7 ± 28.0 (0.0–116.0)
Facial	6.8 ± 7.5 (0.0–35.0)
Verbal	10.9 ± 11.1 (0.0–50.0)
Behavioral	14.0 ± 10.9 (0.0–43.0)

Note: Continuous measures are presented as mean ± standard deviation and range of values. N is the number of patients. VAS is the visual analog scale; BMIS is the brief mood introspection scale; STAI-C is the state–trait anxiety inventory for children; PCS-C is the pain catastrophizing scale for children; APBQ is the adolescent pain behavior questionnaire.

**Table 3:** Correlation (Pearson) between teens’ and parents’ responses—All patients.

N = 202	Parent VAS	Parent APBQ	Parent Facial	Parent Verbal	Parent Behavioral
Teen VAS	**0.56**	**0.16**	**0.17**	**0.16**	0.10
BMIS	**−0.34**	**−0.24**	**−0.20**	**−0.24**	**−0.19**
STAI-C *	**0.39**	**0.27**	**0.21**	**0.29**	**0.23**
PCS-C	**0.28**	**0.25**	**0.17**	**0.23**	**0.25**
Rumination	**0.26**	**0.23**	**0.17**	**0.22**	**0.23**
Magnification *	**0.20**	**0.20**	0.12	**0.19**	**0.21**
Helplessness	**0.27**	**0.23**	**0.17**	**0.21**	**0.24**

Note: Correlation coefficients listed in bold are significant at the 0.05 level. N is the number of patients. * Spearman correlation coefficient. VAS is the visual analog scale; BMIS is the brief mood introspection scale; STAI-C is the state–trait anxiety inventory for children; PCS-C is the pain catastrophizing scale for children.

**Table 4:** Correlation (Spearman) between teens’ and nurses’ responses—All patients.

N = 202	Nurse VAS	Nurse APBQ	Nurse Facial	Nurse Verbal	Nurse Behavioral
Teen VAS	**0.47**	**0.16**	**0.17**	**0.14**	**0.14**
BMIS	**−0.29**	**−0.24**	**−0.25**	**−0.21**	**−0.22**
STAI-C	**0.26**	**0.19**	**0.19**	**0.14**	**0.19**
PCS-C	**0.17**	**0.14**	0.12	**0.15**	0.13
Rumination	**0.23**	0.12	0.11	0.13	0.10
Magnification	**0.15**	**0.14**	0.13	0.11	0.13
Helplessness	0.12	**0.14**	0.12	**0.15**	0.12

Note: Correlation coefficients listed in bold are significant at the 0.05 level. N is the number of patients. VAS is the visual analog scale; BMIS is the brief mood introspection scale; STAI-C is the state–trait anxiety inventory for children; PCS-C is the pain catastrophizing scale for children.

**Table 5:** Correlation (Pearson) between teens’ various scale responses—All patients.

N = 202	BMIS	STAI-C *	PCS-C	Rumination	Magnification *	Helplessness
Teen VAS	**−0.36**	**0.39**	**0.35**	**0.36**	**0.26**	**0.32**
BMIS	---	**−0.84**	**−0.41**	**−0.37**	**−0.30**	**−0.38**
STAI-C *	---	---	**0.40**	**0.36**	**0.35**	**0.36**
PCS-C	---	---	---	**0.88**	**0.80**	**0.95**
Rumination	---	---	---	---	**0.60**	**0.75**
Magnification *	---	---	---	---	---	**0.68**

Note: The correlation coefficients listed in bold are significant at 0.05. N is the number of patients. * Spearman correlation coefficient. VAS is the visual analog scale; BMIS is the brief mood introspection scale; STAI-C is the state–trait anxiety inventory for children; PCS-C is the pain catastrophizing scale for children.

## Data Availability

The original contributions presented in the study are included in the article/Supplementary Material, further inquiries can be directed to the corresponding author.

## Supplementary Materials

The following supporting information can be downloaded at https://www.mdpi.com/article/10.3390/children11040493/s1: Figure S1: CONSORT 2010 Flow Diagram; Table S1: Correlation (Pearson) between teens’ and parents’ responses—Teens age 11–13; Table S2: Correlation (Spearman) between teens’ and nurses’ responses—Teens aged 11–13; Table S3: Correlation (Pearson) between teens’ various scale responses—Teens age 11–13; Table S4: Correlation (Pearson) between teens’ and parents’ responses—Teens age 14–17; Table S5: Correlation (Spearman) between teens’ and nurses’ responses—Teens aged 14–17; Table S6:Correlation (Pearson) between teens’ various scale responses—Teens age 14–17; Table S7: Correlation (Pearson) between teens’ and parents’ responses—Girls; Table S8: Correlation (Spearman) between teens’ and nurses’ responses—Girls; Table S9: Correlation (Pearson) between teens’ various scale responses—Girls; Table S10: Correlation (Pearson) between teens’ and parents’ responses—Boys; Table S11: Correlation (Spearman) between teens’ and nurses’ responses—Boys; Table S12: Correlation (Pearson) between teens’ various scale responses—Boys.
