# Supplementary material for: Gaining Insight into Teenagers’ Experiences of Pain after Laparoscopic Surgeries: A Prospective Study
Source: Children (Basel). 2024 Apr 20;11(4):493. doi: 10.3390/children11040493 (PMC11049025; doi:10.3390/children11040493)
Supplement: Supplementary file 1 [file children-11-00493-s001.zip › children-2907386-supplementary.pdf]

CONSORT
TRANSPARENT REPORTING of TRIALS

**Figure S1: CONSORT 2010 Flow Diagram**

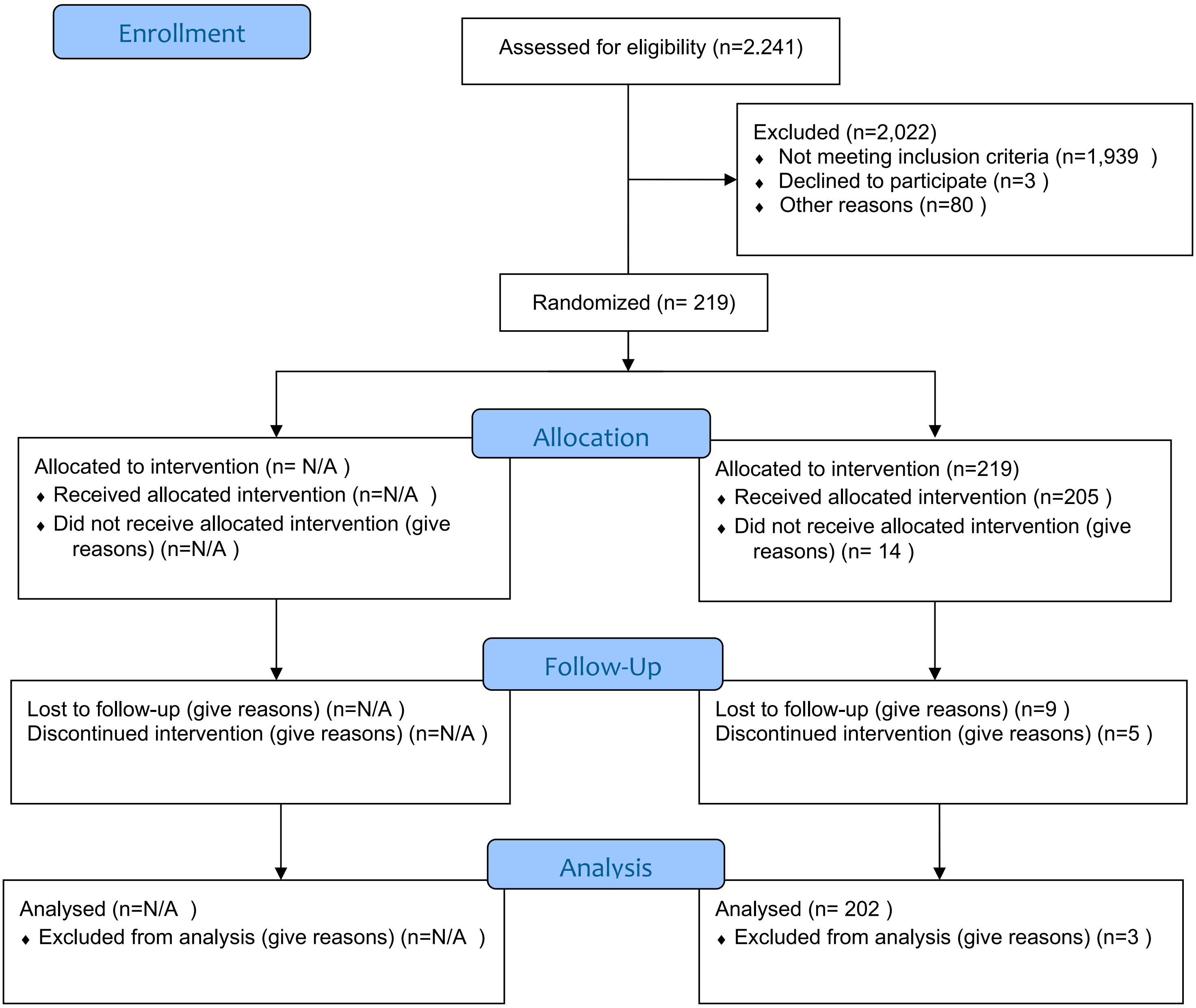

Table S1. Correlation (Pearson) between teens' and parents' responses – Teens age 11-13.

| N = 88 | Parent VAS | Parent APBQ | Parent Facial | Parent Verbal | Parent Behavioral |
|---|---|---|---|---|---|
| Teen VAS | **0.61** | 0.09 | 0.10 | 0.10 | 0.05 |
| BMIS | **-0.47** | **-0.24** | -0.14 | **-0.30** | -0.18 |
| STAI-C | **0.48** | **0.21** | 0.07 | **0.28** | 0.18 |
| PCS-C | 0.19 | 0.07 | -0.05 | 0.08 | 0.13 |
| Rumination | **0.22** | 0.07 | 0.0003 | 0.08 | 0.11 |
| Magnification | 0.15 | 0.02 | -0.11 | 0.04 | 0.11 |
| Helplessness | 0.16 | 0.07 | -0.04 | 0.08 | 0.13 |

Note: Correlation coefficients listed in bold are significant at the 0.05 level. N is the number of patients. VAS is the Visual Analog Scale; BMIS is the Brief Mood Introspection Scale; STAI-C –is the State-Trait Anxiety Inventory for Children; PCS-C is the Pain Catastrophizing Scale for Children.

Table S2. Correlation (Spearman) between teens' and nurses' responses – Teens aged 11-13.

| N = 88 | Nurse VAS | Nurse APBQ | Nurse Facial | Nurse Verbal | Nurse Behavioral |
|---|---|---|---|---|---|
| Teen VAS | **0.40** | **0.23** | **0.25** | **0.24** | **0.21** |
| BMIS | **-0.29** | -0.19 | **-0.21** | **-0.21** | -0.17 |
| STAI-C | 0.19 | 0.17 | 0.17 | 0.17 | 0.16 |
| PCS-C | 0.18 | 0.09 | 0.08 | 0.11 | 0.06 |
| Rumination | 0.18 | 0.06 | 0.09 | 0.09 | 0.03 |
| Magnification | 0.13 | 0.12 | 0.09 | 0.15 | 0.10 |
| Helplessness | 0.14 | 0.07 | 0.06 | 0.08 | 0.06 |

Note: Correlation coefficients listed in bold are significant at the 0.05 level. N is the number of patients. VAS is the Visual Analog Scale; BMIS is the Brief Mood Introspection Scale; STAI-C –is the State-Trait Anxiety Inventory for Children; PCS-C is the Pain Catastrophizing Scale for Children.

Table S3. Correlation (Pearson) between teens' various scale responses – Teens age 11-13.

| N = 88 | BMIS | STAI-C | PCS-C | Rumination | Magnification | Helplessness |
|---|---|---|---|---|---|---|
| Teen VAS | **-0.46** | **0.44** | **0.35** | **0.36** | **0.30** | **0.30** |
| BMIS | --- | **-0.89** | **-0.38** | **-0.37** | **-0.31** | **-0.35** |
| STAI-C | --- | --- | **0.44** | **0.37** | **0.41** | **0.42** |
| PCS-C | --- | --- | --- | **0.90** | **0.84** | **0.95** |
| Rumination | --- | --- | --- | --- | **0.66** | **0.77** |
| Magnification | --- | --- | --- | --- | --- | **0.72** |

Note: Correlation coefficients listed in bold are significant at the 0.05 level. N is the number of patients. VAS is the Visual Analog Scale; BMIS is the Brief Mood Introspection Scale; STAI-C –is the State-Trait Anxiety Inventory for Children; PCS-C is the Pain Catastrophizing Scale for Children.

Table S4. Correlation (Pearson) between teens' and parents' responses – Teens age 14-17.

| N = 114 | Parent VAS | Parent APBQ | Parent Facial | Parent Verbal | Parent Behavioral |
|---|---|---|---|---|---|
| Teen VAS | **0.53** | **0.21** | **0.22** | **0.21** | 0.14 |
| BMIS | **-0.24** | **-0.23** | **-0.25** | **-0.18** | **-0.19** |
| STAI-C | **0.28** | **0.32** | **0.29** | **0.27** | **0.28** |
| PCS-C | **0.35** | **0.38** | **0.32** | **0.35** | **0.33** |
| Rumination | **0.28** | **0.35** | **0.28** | **0.33** | **0.31** |
| Magnification* | **0.24** | **0.28** | **0.25** | **0.27** | **0.24** |
| Helplessness | **0.35** | **0.34** | **0.30** | **0.30** | **0.31** |

Note: Correlation coefficients listed in bold are significant at the 0.05 level. N is the number of patients. *Spearman correlation coefficient, VAS is the Visual Analog Scale; BMIS is the Brief Mood Introspection Scale; STAI-C –is the State-Trait Anxiety Inventory for Children; PCS-C is the Pain Catastrophizing Scale for Children.

Table S5. Correlation (Spearman) between teens' and nurses' responses – Teens aged 14-17.

| N = 114 | Nurse VAS | Nurse APBQ | Nurse Facial | Nurse Verbal | Nurse Behavioral |
|---|---|---|---|---|---|
| Teen VAS | **0.51** | 0.10 | 0.11 | 0.06 | 0.09 |
| BMIS | **-0.31** | **-0.28** | **-0.26** | **-0.21** | **-0.27** |
| STAI-C | **0.32** | **0.22** | **0.22** | 0.12 | **0.24** |
| PCS-C | 0.18 | **0.19** | 0.17 | 0.18 | **0.20** |
| Rumination | **0.27** | 0.16 | 0.12 | 0.16 | 0.16 |
| Magnification | 0.17 | 0.14 | 0.16 | 0.07 | 0.16 |
| Helplessness | 0.12 | **0.18** | 0.17 | **0.19** | 0.17 |

Note: Correlation coefficients listed in bold are significant at the 0.05 level. N is the number of patients. VAS is the Visual Analog Scale; BMIS is the Brief Mood Introspection Scale; STAI-C –is the State-Trait Anxiety Inventory for Children; PCS-C is the Pain Catastrophizing Scale for Children.

Table S6. Correlation (Pearson) between teens' various scale responses – Teens age 14-17.

| N = 114 | BMIS | STAI-C | PCS-C | Rumination | Magnification* | Helplessness |
|---|---|---|---|---|---|---|
| Teen VAS | **-0.28** | **0.39** | **0.36** | **0.36** | **0.23** | **0.33** |
| BMIS | --- | **-0.80** | **-0.44** | **-0.37** | **-0.34** | **-0.42** |

| | | | | | | |
|---|---|---|---|---|---|---|
| STAI-C | --- | --- | **0.47** | **0.37** | **0.39** | **0.43** |
| PCS-C | --- | --- | --- | **0.87** | **0.77** | **0.95** |
| Rumination | --- | --- | --- | --- | **0.54** | **0.73** |
| Magnification* | --- | --- | --- | --- | --- | **0.66** |

Note: Correlation coefficients listed in bold are significant at the 0.05 level. N is the number of patients. *Spearman correlation coefficient. VAS is the Visual Analog Scale; BMIS is the Brief Mood Introspection Scale; STAI-C –is the State-Trait Anxiety Inventory for Children; PCS-C is the Pain Catastrophizing Scale for Children.

Table S7. Correlation (Pearson) between teens' and parents' responses – Girls.

| N = 114 | Parent VAS | Parent APBQ | Parent Facial | Parent Verbal | Parent Behavioral |
|---|---|---|---|---|---|
| Teen VAS | **0.56** | 0.16 | **0.25** | 0.13 | 0.05 |
| BMIS | **-0.41** | **-0.24** | **-0.22** | **-0.26** | -0.16 |
| STAI-C | **0.37** | **0.26** | **0.20** | **0.28** | **0.19** |
| PCS-C | **0.23** | **0.20** | 0.11 | 0.18 | **0.21** |
| Rumination | **0.24** | **0.20** | 0.11 | 0.18 | **0.22** |
| Magnification | 0.17 | **0.18** | 0.13 | 0.18 | 0.16 |
| Helplessness | **0.20** | 0.15 | 0.08 | 0.13 | 0.18 |

Note: Correlation coefficients listed in bold are significant at the 0.05 level. N is the number of patients. VAS is the Visual Analog Scale; BMIS is the Brief Mood Introspection Scale; STAI-C –is the State-Trait Anxiety Inventory for Children; PCS-C is the Pain Catastrophizing Scale for Children.

Table S8. Correlation (Spearman) between teens' and nurses' responses – Girls.

| N = 114 | Nurse VAS | Nurse APBQ | Nurse Facial | Nurse Verbal | Nurse Behavioral |
|---|---|---|---|---|---|
| Teen VAS | **0.45** | 0.09 | 0.09 | 0.06 | 0.07 |
| BMIS | **-0.25** | **-0.20** | **-0.20** | -0.17 | **-0.19** |
| STAI-C | **0.18** | 0.10 | 0.11 | 0.06 | 0.10 |
| PCS-C | 0.04 | 0.09 | 0.08 | 0.07 | 0.08 |
| Rumination | **0.18** | 0.10 | 0.07 | 0.07 | 0.10 |
| Magnification | 0.01 | 0.04 | 0.06 | 0.02 | 0.02 |

| | | | | | |
|---|---|---|---|---|---|
| Helplessness | 0.004 | 0.09 | 0.09 | 0.08 | 0.07 |

Note: Correlation coefficients listed in bold are significant at the 0.05 level. N is the number of patients. VAS is the Visual Analog Scale; BMIS is the Brief Mood Introspection Scale; STAI-C –is the State-Trait Anxiety Inventory for Children; PCS-C is the Pain Catastrophizing Scale for Children.

Table S9. Correlation (Pearson) between teens' various scale responses – Girls.

| N = 114 | BMIS | STAI-C* | PCS-C | Rumination | Magnification | Helplessness |
|---|---|---|---|---|---|---|
| Teen VAS | **-0.39** | **0.35** | **0.24** | **0.30** | 0.09 | **0.21** |
| BMIS | --- | **-0.85** | **-0.41** | **-0.40** | **-0.32** | **-0.35** |
| STAI-C | --- | --- | **0.40** | **0.35** | **0.38** | **0.34** |
| PCS-C | --- | --- | --- | **0.86** | **0.77** | **0.94** |
| Rumination | --- | --- | --- | --- | **0.53** | **0.71** |
| Magnification | --- | --- | --- | --- | --- | **0.62** |

Note: Correlation coefficients listed in bold are significant at the 0.05 level. N is the number of patients. VAS is the Visual Analog Scale; BMIS is the Brief Mood Introspection Scale; STAI-C –is the State-Trait Anxiety Inventory for Children; PCS-C is the Pain Catastrophizing Scale for Children. * Spearman correlation coefficient.

Table S10. Correlation (Pearson) between teens' and parents' responses – Boys.

| N = 88 | Parent VAS | Parent APBQ | Parent Facial | Parent Verbal | Parent Behavioral |
|---|---|---|---|---|---|
| Teen VAS | **0.56** | 0.11 | 0.04 | 0.14 | 0.12 |
| BMIS | **-0.28** | **-0.25** | -0.18 | **-0.25** | **-0.24** |
| STAI-C | **0.38** | **0.26** | 0.16 | **0.27** | **0.27** |
| PCS-C | **0.32** | **0.23** | 0.18 | **0.21** | **0.24** |
| Rumination | **0.25** | 0.19 | 0.16 | 0.17 | 0.18 |
| Magnification* | **0.22** | 0.16 | 0.10 | 0.15 | 0.19 |
| Helplessness | **0.35** | **0.26** | **0.22** | **0.22** | **0.27** |

Note: Correlation coefficients listed in bold are significant at the 0.05 level. N is the number of patients. *Spearman correlation coefficient. VAS is the Visual Analog Scale; BMIS is the Brief Mood Introspection Scale; STAI-C –is the State-Trait Anxiety Inventory for Children; PCS-C is the Pain Catastrophizing Scale for Children.

Table S11. Correlation (Spearman) between teens' and nurses' responses – Boys.

| N = 88 | Nurse VAS | Nurse APBQ | Nurse Facial | Nurse Verbal | Nurse Behavioral |
|---|---|---|---|---|---|
| Teen VAS | **0.52** | **0.28** | **0.30** | **0.25** | **0.25** |
| BMIS | **-0.35** | **-0.27** | **-0.30** | **-0.25** | **-0.24** |
| STAI-C | **0.36** | **0.30** | **0.32** | **0.22** | **0.30** |
| PCS-C | **0.36** | **0.23** | **0.21** | **0.23** | 0.20 |
| Rumination | **0.35** | 0.15 | 0.16 | 0.17 | 0.10 |
| Magnification | **0.34** | **0.27** | **0.24** | **0.22** | **0.27** |
| Helplessness | **0.30** | **0.23** | 0.20 | **0.24** | 0.21 |

Note: Correlation coefficients listed in bold are significant at the 0.05 level. N is the number of patients. VAS is the Visual Analog Scale; BMIS is the Brief Mood Introspection Scale; STAI-C –is the State-Trait Anxiety Inventory for Children; PCS-C is the Pain Catastrophizing Scale for Children.

Table S12. Correlation (Pearson) between teens' various scale responses – Boys.

| N = 88 | BMIS | STAI-C | PCS-C | Rumination | Magnification* | Helplessness |
|---|---|---|---|---|---|---|
| Teen VAS | **-0.34** | **0.49** | **0.46** | **0.39** | **0.39** | **0.44** |
| BMIS | --- | **-0.85** | **-0.43** | **-0.35** | **-0.29** | **-0.44** |

| | | | | | | |
|---|---|---|---|---|---|---|
| STAI-C | --- | --- | **0.50** | **0.37** | **0.35** | **0.52** |
| PCS-C | --- | --- | --- | **0.90** | **0.85** | **0.96** |
| Rumination | --- | --- | --- | --- | **0.66** | **0.79** |
| Magnification* | --- | --- | --- | --- | --- | **0.75** |

Note: Correlation coefficients listed in bold are significant at the 0.05 level. N is the number of patients. *Spearman correlation coefficient. VAS is the Visual Analog Scale; BMIS is the Brief Mood Introspection Scale; STAI-C –is the State-Trait Anxiety Inventory for Children; PCS-C is the Pain Catastrophizing Scale for Children.

The questionnaires and the score documents used: Visual Analog Scale from the teenager (tVAS ), Visual Analog Scale from the parent (pVAS ), Visual Analog Scale from the nurse (nVAS ), Adolescent Pain Behavior Questionnaire from the parent (pAPBQ), Adolescent Pain Behavior Questionnaire from the nurse (nAPBQ), State-Trait Anxiety Inventory for the children (STAIC S – Anxiety), Pain Catastrophizing Scale for the children (PCS-C), and Brief Mood Introspection Scale (BMIS).

**Pain Questionnaire - Patient**

How severe is your pain now?

Place a vertical mark on the line below to show how much pain you are feeling right now.

**No pain** 0 |————————————————| 100 **The worst pain imaginable**

Fig 1 100 mm Visual Analog Scales **TOTAL SCORE**:

**Pain Questionnaire - Parent**

How severe do you think your child's pain is right now?

Place a vertical mark on the line below to indicate how much pain your child is feeling right now.

**No pain** 0 |————————————————| 100 **The worst pain imaginable**

Fig 1 100 mm Visual Analog Scales **TOTAL SCORE:**

**Pain Questionnaire - Nurse**

How severe do you think your patient's pain is right now?

Place a vertical mark on the line below to indicate how much pain your patient is feeling right now.

**No pain** 0 |————————————————| 100 **The worst pain imaginable**

Fig 1 100 mm Visual Analog Scales

**TOTAL SCORE:**

**Subject #: ________**

**Date: ___ ____________ _______**

**Time: __ __:__ __**

**Adolescent Pain Behaviors Questionnaire- Parent**

Below is a list of common ways that children and teenagers use their faces to express when they are in pain. Please rate each behavior from 0 (Never) to 5 (Almost Always) to show how often you notice your child making these facial responses when he/she is experiencing pain.

| | | N ever | Almost Never | Some times | Fairly Often | ( ften | Almost Always |
|---|---|---|---|---|---|---|---|
| . | Face changes color (red, pale) | 0 | 1 | 2 | 3 | [illegible] | 5 |
| . | Dazed/eyes glazed | 0 | 1 | 2 | 3 | [illegible] | 5 |
| . | Clenched jaw | 0 | 1 | 2 | 3 | [illegible] | 5 |
| . | Frowning | 0 | 1 | 2 | 3 | [illegible] | 5 |
| . | Circles under eyes | 0 | 1 | 2 | 3 | [illegible] | 5 |
| . | Wincing/grimacing | 0 | 1 | 2 | 3 | [illegible] | 5 |

| | | | | | | | |
|---|---|---|---|---|---|---|---|
| . | Tears in eyes | 0 | 1 | 2 | 3 | 4 | 5 |

Below is a list of common things that children and teenagers may say or do when they are in pain. Please rate each behavior from 0 (Never) to 5 (Almost Always) to show how often you notice your child making these sounds or asking these questions when he/she is experiencing pain.

| | | N<br>ever | Almost<br>Never | Someti<br>mes | Fairly<br>Often | Oft<br>en | Almost<br>Always |
|---|---|---|---|---|---|---|---|
| . | Whines | 0 | 1 | 2 | 3 | 4 | 5 |
| . | Complains/talks about hurting | 0 | 1 | 2 | 3 | 4 | 5 |
| 0. | Cries | 0 | 1 | 2 | 3 | 4 | 5 |
| 1. | Sighs | 0 | 1 | 2 | 3 | 4 | 5 |
| 2. | Groans | 0 | 1 | 2 | 3 | 4 | 5 |
| 3. | Asks Mom or Dad for help | 0 | 1 | 2 | 3 | 4 | 5 |
| 4. | Whimpers | 0 | 1 | 2 | 3 | 4 | 5 |
| 5. | Yells or screams when in pain | 0 | 1 | 2 | 3 | 4 | 5 |
| 6. | Gets irritable/moody | 0 | 1 | 2 | 3 | 4 | 5 |
| 7. | Gets quiet | 0 | 1 | 2 | 3 | 4 | 5 |

**Subject #: ________**

Below is a list of things that children and teenagers may do when they are in pain. Please rate the behaviors from 0 (Never) to 5 (Almost Always) to show how often you notice your child making these actions and gestures when he/she is experiencing pain.

| | | N<br>ever | Almost<br>Never | Some<br>times | Fairly<br>Often | (<br>ften | Almost<br>Always |
|---|---|---|---|---|---|---|---|
| 18. | Fidgeting or restless | C | 1 | 2 | 3 | 4 | 5 |
| 19. | Tense body | C | 1 | 2 | 3 | 4 | 5 |
| 20. | Hunched over or stooping | C | 1 | 2 | 3 | 4 | 5 |
| 21. | Holding area of body that hurts | C | 1 | 2 | 3 | 4 | 5 |
| 22. | Moves slowly or protectively | C | 1 | 2 | 3 | 4 | 5 |
| 23. | Walks with a limp | C | 1 | 2 | 3 | 4 | 5 |
| 24. | Rubbing area of body that hurts | C | 1 | 2 | 3 | 4 | 5 |
| 25. | Avoids touching/bumping area of body that hurts | C | 1 | 2 | 3 | 4 | 5 |
| 26. | Flinches or jerks when painful area is touched | C | 1 | 2 | 3 | 4 | 5 |

Other comments regarding your child's behavior:

________________________________________________________________

________________________________________________________________

________________________________________________________________

___________________________________________________________________________________

_________________________________________________________________________________

____________________________

**<u>TOTAL SCORE</u>:**

This questionnaire was adapted from the Adolescent Pain Behavior Questionnaire described in the 2010 article from the PAIN journal Vol. 151, 834-842, “Parent perceptions of adolescent pain expression: The adolescent pain behavior questionnaire,” by A.M. Lynch-Jordan, S. Kashikar-Zuck, and K.R. Goldschneider.

**Subject #: ________**

**Date: ___ _____________ ________**

**Time: __ __:__ __**

**Adolescent Pain Behaviors Questionnaire- Nurse**

Below is a list of common ways that children and teenagers use their faces to express when they are in pain. Please rate each behavior from 0 (Never) to 5 (Almost Always) to show how often you notice your patient making these facial responses when he/she is experiencing pain.

| | Never | Almost Never | Sometimes | Fairly Often | Often | Almost Always |
|---|---|---|---|---|---|---|
| . Face changes color (red, pale) | 0 | 1 | 2 | 3 | 4 | 5 |

| | | | | | | | |
|---|---|---|---|---|---|---|---|
| . | Dazed/eyes glazed | 0 | 1 | 2 | 3 | 4 | 5 |
| . | Clenched jaw | 0 | 1 | 2 | 3 | 4 | 5 |
| . | Frowning | 0 | 1 | 2 | 3 | 4 | 5 |
| . | Circles under eyes | 0 | 1 | 2 | 3 | 4 | 5 |
| . | Wincing/grimacing | 0 | 1 | 2 | 3 | 4 | 5 |
| . | Tears in eyes | 0 | 1 | 2 | 3 | 4 | 5 |

Below is a list of common things that children and teenagers may say or do when they are in pain. Please rate each behavior from 0 (Never) to 5 (Almost Always) to show how often you notice your patient making these sounds or asking these questions when he/she is experiencing pain.

| | | N ever | Almost Never | Someti mes | Fairly Often | Oft en | Almost Always |
|---|---|---|---|---|---|---|---|
| . | Whines | 0 | 1 | 2 | 3 | 4 | 5 |
| . | Complains/talks about hurting | 0 | 1 | 2 | 3 | 4 | 5 |
| 0. | Cries | 0 | 1 | 2 | 3 | 4 | 5 |
| 1. | Sighs | 0 | 1 | 2 | 3 | 4 | 5 |
| 2. | Groans | 0 | 1 | 2 | 3 | 4 | 5 |
| 3. | Asks Mom or Dad for help | 0 | 1 | 2 | 3 | 4 | 5 |
| 4. | Whimpers | 0 | 1 | 2 | 3 | 4 | 5 |
| 5. | Yells or screams when in pain | 0 | 1 | 2 | 3 | 4 | 5 |
| 6. | Gets irritable/moody | 0 | 1 | 2 | 3 | 4 | 5 |
| 7. | Gets quiet | 0 | 1 | 2 | 3 | 4 | 5 |

**Subject #: ________**

Below is a list of things that children and teenagers may do when they are in pain. Please rate the behaviors from 0 (Never) to 5 (Almost Always) to show how often you notice your patient making these actions and gestures when he/she is experiencing pain.

| | | N ever | Almost Never | Some times | Fairly Often | O ften | Almost Always |
|---|---|---|---|---|---|---|---|
| 18. | Fidgeting or restless | 0 | 1 | 2 | 3 | 4 | 5 |
| 19. | Tense body | 0 | 1 | 2 | 3 | 4 | 5 |
| 20. | Hunched over or stooping | 0 | 1 | 2 | 3 | 4 | 5 |
| 21. | Holding area of body that hurts | 0 | 1 | 2 | 3 | 4 | 5 |

| | | | | | | | |
|---|---|---|---|---|---|---|---|
| 22. | Moves slowly or protectively | 0 | 1 | 2 | 3 | 4 | 5 |
| 23. | Walks with a limp | 0 | 1 | 2 | 3 | 4 | 5 |
| 24. | Rubbing area of body that hurts | 0 | 1 | 2 | 3 | 4 | 5 |
| 25. | Avoids touching/bumping area of body that hurts | 0 | 1 | 2 | 3 | 4 | 5 |
| 26. | Flinches or jerks when painful area is touched | 0 | 1 | 2 | 3 | 4 | 5 |

Other comments regarding your patient's behavior:

**<u>TOTAL SCORE</u>**:

This questionnaire was adapted from the Adolescent Pain Behavior Questionnaire described in the 2010 article from the PAIN journal Vol. 151, 834-842, "Parent perceptions of adolescent pain expression: The adolescent pain behavior questionnaire," by A.M. Lynch-Jordan, S. Kashikar-Zuck, and K.R. Goldschneider.

**HOW-I-FEEL QUESTIONNAIRE sample**

Developed by C.D. Spielberger, C.D. Edwards, J. Montuori, and R. Lushene

STAIC Form C-1:
SUBJECT # ____________________________ Date:_________ Time:

DIRECTIONS: A number of statements which boys and girls use to describe themselves are given below. Read each statement carefully and decide how you feel *right now.* Then put an X in the box in front of the word or phrase which best describes how you feel. There are no right or wrong answers. Don't spend too much time on any one statement. Remember, find the word or phrase which best describes how you feel right now, *at this very moment*.

1. I feel .......................................... very calm calm not calm
2. I feel .......................................... very upset upset not upset
3. I feel .......................................... very pleasant pleasant not pleasant
4. I feel .......................................... very nervous nervous not nervous
5. I feel .......................................... very jittery jittery not jittery
6. I feel .......................................... very rested rested not rested
7. I feel .......................................... very scared scared not scared
8. I feel .......................................... very relaxed relaxed not relaxed
9. I feel .......................................... very worried worried not worried
10. I feel .......................................... very satisfied satisfied not satisfied
11. I feel .......................................... very frightened frightened not frightened
12. I feel .......................................... very happy happy not happy
13. I feel .......................................... very sure sure not sure
14. I feel .......................................... very good good not good
15. I feel .......................................... very troubled troubled not troubled
16. I feel .......................................... very bothered bothered not bothered
17. I feel .......................................... very nice nice not nice
18. I feel .......................................... very terrified terrified not terrified
19. I feel .......................................... very mixed-up mixed-up not mixed-up
20. I feel .......................................... very cheerful cheerful not cheerful

Scoring Key for STAI for Children Sample

**Scoring Instructions for STAIC Form C-1**

Fold this paper in half and line up next to the appropriate item numbers on the answer sheet. Be sure you are on the correct side of the answer sheet (Form C-1). Total the scoring weights shown for the marked responses.

1. .......................................... 1 2 3
2. .......................................... 3 2 1
3. .......................................... 1 2 3
4. .......................................... 3 2 1
5. ........................................ 3 2 1
6. .......................................... 1 2 3
7. .......................................... 3 2 1
8. .......................................... 1 2 3
9. .......................................... 3 2 1
10. .......................................... 1 2 3

11. .......................................... 3 2 1
12. .......................................... 1 2 3
13. .......................................... 1 2 3
14. .......................................... 1 2 3
15. .......................................... 3 2 1
16. .......................................... 3 2 1
17. .......................................... 1 2 3
18. .......................................... 3 2 1
19. .......................................... 3 2 1
20. .......................................... 1 2 3

Total Score for C-1 _______________

**Subject #: ________**
**Date: ___ ________ ________**
**Time: ___ ___:___ ___**

**Thoughts and Feelings During Pain (PCS-C)**

We are interested in what you think and how strong the feelings are when you are in pain. Below are 13 different thoughts and feelings you may have when you are in pain. On a scale from 0 (Not at all) to 4 (Extremely), try to show us as clearly as possible what you think and feel by putting a circle around the word that best reflects how strongly you have each thought, after each sentence.

| | | **Not at all** | **Mildly** | **Moderately** | **Severely** | **Extremely** |
|---|---|---|---|---|---|---|
| 1. | When I am in pain, I worry all the time about whether the pain will end. | 0 | 1 | 2 | 3 | 4 |
| 2. | When I am in pain, I feel I can't go on like this much longer. | 0 | 1 | 2 | 3 | 4 |
| 3. | When I am in pain, it's terrible and I think it's never going to get better. | 0 | 1 | 2 | 3 | 4 |
| 4. | When I am in pain, it's awful and I feel that it takes over me. | 0 | 1 | 2 | 3 | 4 |
| 5. | When I am in pain, I can't stand it anymore. | 0 | 1 | 2 | 3 | 4 |
| 6. | When I am in pain, I become afraid that the pain will get worse. | 0 | 1 | 2 | 3 | 4 |
| 7. | When I am in pain, I keep thinking of other painful events. | 0 | 1 | 2 | 3 | 4 |
| 8. | When I am in pain, I want the pain to go away. | 0 | 1 | 2 | 3 | 4 |
| 9. | When I am in pain, I can't keep it out of my mind. | 0 | 1 | 2 | 3 | 4 |
| 10. | When I am in pain, I keep thinking about how much it hurts. | 0 | 1 | 2 | 3 | 4 |
| 11. | When I am in pain, I keep thinking about how much I want the pain to stop. | 0 | 1 | 2 | 3 | 4 |
| 12. | When I am in pain, there is nothing I can do to stop the pain. | 0 | 1 | 2 | 3 | 4 |
| 13. | When I am in pain, I wonder whether something serious may happen. | 0 | 1 | 2 | 3 | 4 |

**<u>TOTAL SCORE:</u>**

This questionnaire was adapted from the pain catastrophizing scale for children (PCS-C) described in the 2003 publication in the PAIN journal, Vol. 104, 639-646, “The child version of the pain catastrophizing scale (PCS-C): a preliminary validation,” by G. Crombez, P. Bijttebier, C. Eccleston, T. Mascagni, G. Mertens, L. Goubert, K. Verstraeten.

Scoring Key for STAI for Children    Sample

**Scoring Instructions for STAIC Form C-1**
Fold this paper in half and line up next to the appropriate item numbers on the answer sheet. Be sure you are on the correct side of the answer sheet (Form C-1). Total the scoring weights shown for the marked responses.

1. .......................................... 1 2 3
2. .......................................... 3 2 1
3. .......................................... 1 2 3
4. .......................................... 3 2 1
5. ........................................ 3 2 1
6. .......................................... 1 2 3
7. .......................................... 3 2 1
8. .......................................... 1 2 3
9. .......................................... 3 2 1
10. .......................................... 1 2 3
11. .......................................... 3 2 1
12. .......................................... 1 2 3
13. .......................................... 1 2 3
14. .......................................... 1 2 3
15. .......................................... 3 2 1
16. .......................................... 3 2 1
17. .......................................... 1 2 3
18. .......................................... 3 2 1
19. .......................................... 3 2 1
20. .......................................... 1 2 3

Total Score for C-1 _______________

**Subject #: ________**

**Date: ___ ____________ _______**

**Time: __ __:__ __**

**Brief Mood Introspection Scale**

INSTRUCTIONS: Circle the response on the scale below that indicates how well each adjective or phrase describes your present mood.

| | | Definitely Do Not Feel | Do Not Feel | Slightly Feel | Definitely Feel |
|---|---|---|---|---|---|
| 1. | Lively | XX | X | V | V.V. |
| 2. | Happy | XX | X | V | V.V. |
| 3. | Sad | XX | X | V | V.V. |
| 4. | Tired | XX | X | V | V.V. |
| 5. | Caring | XX | X | V | V.V. |
| 6. | Content | XX | X | V | VV |
| 7. | Gloomy | XX | X | V | V.V. |
| 8. | Jittery | XX | X | V | V.V. |
| 9. | Drowsy | XX | X | V | V.V. |
| 10. | Grouchy | XX | X | V | VV |
| 11. | Peppy | XX | X | V | V.V. |
| 12. | Nervous | XX | X | V | V.V. |
| 13. | Calm | XX | X | V | V.V. |
| 14. | Loving | XX | X | V | V.V. |
| 15. | Fed up | XX | X | V | V.V. |
| 16. | Active | XX | X | V | VV |

**TOTAL SCORE**:

This questionnaire was adapted from the Brief Introspection Scale (BMIS) published in the Journal of Personality and Social Psychology 1988, Vol. 55, No1, 102-111, "The Experience and Meta-Experience of Mood," by J.D. Mayer and Y.N. Geschke.

**BMIS Scoring- Subtracting**

1. Convert the Meddis response scale (XX, X, V, V.V.) to numbers:

XX = 1
X = 2
V = 3
VV = 4

**Pleasant-Unpleasant Scale**

- Effective range: 24 to -24

**Add:**

- Active

- Calm
- Caring
- Content
- Happy
- Lively
- Loving
- Peppy

**Subtract:**

- Drowsy
- fed up
- gloomy
- grouchy
- jittery
- nervous
- sad
- tired
